# Effects of combined cyclosporin and azithromycin treatment on human mononuclear cells under lipopolysaccharide challenge

**DOI:** 10.3389/froh.2025.1544821

**Published:** 2025-03-13

**Authors:** Norah Alotaibi, Aminah Alesawy, Marwa Alalshaikh, Faisal E. Aljofi, Nada Aldossary, Nada Alzahrani, Omar Omar, Marwa Madi

**Affiliations:** ^1^ Department of Preventive Dental Sciences, College of Dentistry, Imam Abdulrahman Bin Faisal University, Dammam, Saudi Arabia; ^2^ Department of Pathology, College of Medicine, Imam Abdulrahman Bin Faisal University, Dammam, Saudi Arabia; ^3^ Blood Bank, Laboratory Medicine, King Fahad University Hospital, Al Khobar, Saudi Arabia; ^4^ Department of Biomedical Dental Sciences, College of Dentistry, Imam Abdulrahman Bin Faisal University, Dammam, Saudi Arabia

**Keywords:** immunosuppressive agents, interleukin-6 (IL-6), interleukin-1beta (IL-1ß), interleukin-18 (IL-18), immunoglobulin a (IgA)

## Abstract

**Objective:**

To evaluate the combined effects of azithromycin and varying concentrations of cyclosporin on peripheral blood mononuclear cells (PBMCs) under lipopolysaccharide (LPS) stimulation.

**Materials and methods:**

PBMCs were isolated from four healthy donors and treated with cyclosporin at concentrations of (50, 200, and 1,000 ng/ml) either alone or in combination with azithromycin (0.4 µg/ml), with and without 100 ng ml LPS derived from Porphyromonas gingivalis. Total cell count, cell viability, and lactate dehydrogenase (LDH) activity were assessed at day 1 and 3. While the inflammatory mediators, including IL-6, IL-1β, IL-18, and IgA levels were assessed by ELISA at day 3. Statistical analysis included two-way ANOVA to analyze the effects of the drugs and the presence of LPS (the two independent variables), followed by Tukey's HSD *post-hoc* test. Multiple linear regression models evaluating treatment effects, LPS exposure, and time points, with assessment of two-way interactions. Models were adjusted for relevant covariates and verified for statistical assumptions, with significance set at *p* < 0.05.

**Results:**

Lower cyclosporin concentrations (50 and 200 ng/ml) combined with azithromycin maintained higher cell counts and showed reduced cytotoxicity compared to 1,000 ng/ml under LPS exposure. The 200 ng/ml cyclosporin-azithromycin combination demonstrated optimal results, reducing IL-6 and IL-1β levels while maintaining cell viability. Higher concentrations elevated IgA levels, particularly with LPS stimulation, suggesting enhanced immune response modulation.

**Conclusion:**

The combination of azithromycin with moderate cyclosporin concentrations (200 ng/ml) provides optimal immunomodulatory effects while maintaining cell viability. Higher cyclosporin doses (1,000 ng/ml) showed increased cytotoxicity despite enhanced immunomodulation.

## Introduction

Immunosuppressants are a type of medication that is widely used in patients with autoimmune disorders and post-organ transplants to prevent organ rejection (1). Cyclosporine, a calcineurin inhibitor, acts as an immunosuppressant by inhibiting signal transduction via the T cell receptor (2). While cyclosporine has significantly improved survival rates for organ transplants, it is associated with multiple side effects, including nephrotoxicity, neurotoxicity, hypertension, and an increased risk of cardiovascular disease (1) Gingival enlargement has also been reported as a side effect since the first clinical trials of cyclosporine (1, 3). The terms “gingival enlargement” or “gingival overgrowth” are used to describe gingival lesions related to medications, previously referred to as “gingival hyperplasia” or “gingival hypertrophy” (4).

Immunosuppressant drugs can cause gingival enlargement through a direct mechanism involving the excessive buildup of the extracellular matrix, as well as cellular hyperplasia and hypertrophy (5). Additionally, they may act indirectly by modifying the immune response and the activity of inflammatory cells. Previous studies (5, 6) suggested that cyclosporine may both inhibit collagen synthesis and accumulation and intensify the innate immune response.

Although cyclosporine alone does not trigger significant inflammatory responses, it suppresses IL-2-dependent T lymphocyte clonal expansion (7). Additionally, it enhances the expression of CD54, which facilitates the recruitment of pro-inflammatory cells, and increases the production of IL-6 and IL-8 when stimulated by toll-like receptor 2 (TLR2) or toll-like receptor 4 (TLR4) ligands (6). Cyclosporine influences the innate immune response by reducing the release of pro-inflammatory cytokines such as IL-2, IL-12, and IL-10 by macrophages. However, it indirectly promotes an increase in IL-6 and IL-8 secretion through its effects on gingival fibroblasts (2, 6).

Azithromycin, a macrolide antibiotic derived from erythromycin, exerts a bacteriostatic effect on a wide range of Gram-positive and Gram-negative bacteria by interfering with protein synthesis and inhibiting mRNA translation (8). In addition to its antimicrobial properties, azithromycin has significant immunomodulatory effects and has been reported to inhibit gingival enlargement induced by cyclosporine and nifedipine (8). Azithromycin has been shown to decrease the production of IL-6, IL-12, IL-1β and TNF-α (9–11) as well as stimulate the production of IL-10 by monocytes and macrophages (9, 12). Although azithromycin inhibits various pro-inflammatory pathways, it does not lead to full immune suppression as glucocorticoids and other immunosuppressive drugs. Rather, azithromycin exhibits immunomodulatory effect by shifting the inflammatory response of macrophages, toward regulation and tissue repair (13, 14).

Both cyclosporin and azithromycin have significant immunomodulatory effects. Cyclosporin is known to suppress the immune system, primarily affecting T lymphocytes, while azithromycin has anti-inflammatory properties (5, 15). Previous animal studies have (16, 17) demonstrated that azithromycin reduces gingival enlargement and increases MMP-1 expression, which promotes the degradation of excessive collagen and improves cyclosporine-induced gingival overgrowth by inhibiting cell proliferation and collagen synthesis. Macrophage has two main phenotypes, pro-inflammatory and anti-inflammatory which are commonly described as M1 and M2, respectively. Pro-inflammatory M1 phenotype, release several inflammatory mediators including IL-1β (18).

*In vitro* studies (19, 20) have often used a low concentration of cyclosporin A (10 ng/ml), which may have contributed to the reported positive effects. Our study aims to address this by using different concentrations of cyclosporin that more closely resemble the drug plasma concentrations used clinically. Additionally, we include the presence of bacterial lipopolysaccharides (LPS) to better simulate clinical scenarios. Drug-induced gingival overgrowth occurs as a side effect following the systemic administration of drugs used for treatment of various medical particularly immunosuppressive drugs. Although the beneficial effect of these immunosuppressive drugs for patients, gingival enlargement can significantly impact aesthetics, function, speech, and oral hygiene maintenance, compromising the overall quality of life (21). Studying the combined effects of cyclosporin and azithromycin on mononuclear cells can provide a valuable understanding on how these drugs interact at the cellular level, particularly in an immune context. Different concentrations of cyclosporin may have varying impacts on function and viability. By evaluating these effects in combination with azithromycin, it is possible to determine the optimal dosing regimen that maximizes therapeutic benefits while minimizing adverse effects. This is particularly important for patients who require both immunosuppressive and anti-inflammatory treatment.

Thus, the aim of this study was to evaluate the effect of different cyclosporin concentrations combined with azithromycin on peripheral blood human mononuclear cells in the presence and absence of bacterial lipopolysaccharides.

## Material and methods

In this *in vitro* study, the effect of different cyclosporin concentrations combined with azithromycin on PBMC with and without LPS was evaluated. Written informed consent was obtained from all participants at the time of blood donation. The study was conducted in compliance with the Declaration of Helsinki and received ethical approval from the Institutional Review Board at Imam Abdulrahman bin Faisal University, Dammam, Saudi Arabia (IRB-PGS-2023-02-508).

### Peripheral blood mononuclear cells isolation

Using the Ficoll-Paque PREMIUM 1.073 (Cytiva, formerly GE Healthcare Life Sciences, Marlborough, MA, USA) separation gradient technique, mononuclear cells were extracted separately from 1-day-old buffy coats. Two buffy coats were obtained from each of the four individual healthy donors at the blood bank (*n* = 8). Each buffy coat was first mixed and diluted with an equal volume of Hanks' balanced salt solution (HBSS). Then, 4 ml of each diluted buffy coat sample was carefully layered in a separate conical tube on top of 3 ml of the Ficoll-Paque solution and subsequently centrifuged at 400 g for 40 min at 20°C

Thereafter, the layers of mononuclear cells were carefully aspirated and transferred to a sterile centrifuge tube using a sterile pipette. The transferred cells were re-suspended in HBSS and washed twice by repeated centrifugation at 500 × g for 15 min at 20°C.

After the last centrifugation, the supernatants were removed, and the cells were counted using an automated NucleoCounter (NucleoCounter NC202; hemoMetec). The cells were diluted to the required concentration (500,000 cells/ml) in complete culture media [RPMI containing 5% fetal bovine serum (FBS), 1% Penicillin-Streptomycin and 1% L-glutamine; Sigma]. The cells were seeded in the 24-well plates (500,000 cells/ml) for receiving the different medications. Half of the plates were exposed to the same concentrations of 100 ng ml LPS (Porphyromonas gingivalis LPS, InvivoGen). LPS was provided in a lyophilized (freeze-dried) form, and was reconstituted in cell culture medium at1 mg/ml before being used in the experiments at a concentration of 100 ng/ml. After 24- and 72-h incubation period the conditioned medium from each well was harvested and immediately frozen and stored at −70°C until analyzed by polyclonal sandwich ELISAs according to the manufacturer's instructions.

### Medication preparation and study groups

**Figure 1:**
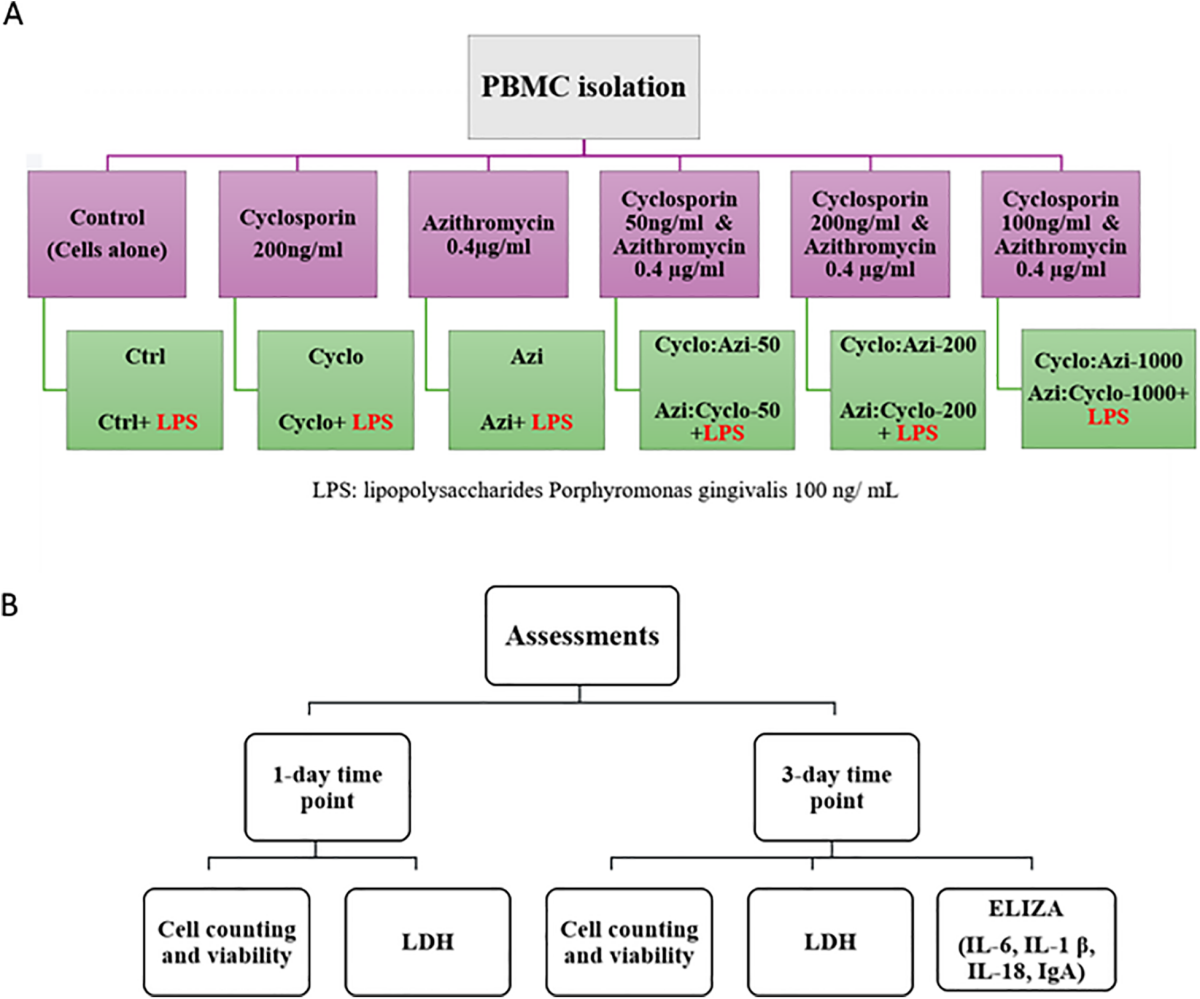
**(A)** Flow chart showing the study groups and **(B)** summary of assessments at day 1 and day 3.

The study utilized commercially available drugs: azithromycin (Riyadh Pharma), which was dissolved and diluted in dimethyl sulfoxide (DMSO) to achieve a concentration of 0.4 mg/ml, and cyclosporin A (Novartis), also dissolved in DMSO to create concentrations of 50 ng/ml, 200 ng/ml, and 1000 ng/ml. The experiment included 12 groups with a sample size of 8 in each group (4 biological × 2 technical = 8) to examine the effect of the two drugs: no medication groups (Ctrl): PBMC were incubated in medium with and without LPS. cyclosporin groups (Cyclo): PBMC were treated with cyclosporin at a 200 ng/ml concentration, with and without LPS. Azithromycin groups (Azi): PBMC were treated with azithromycin at a 0.4 µg/ml concentration, with and without LPS. Combined azithromycin/cyclosporin groups (Azi:Cyclo): PBMC were treated with 0.4 µg/ml azithromycin in combination with cyclosporin at concentrations of 50 ng/ml, 200 ng/ml, and 1,000 ng/ml, with and without LPS (Figure 1A).

### Total cells count and viability percentage analysis: (12 groups × 8 samples/group × 2 time points)

After collecting the culture supernatant, the wells were gently washed twice with PBS to remove nonadherent nonviable cells. The cells were then detached with 0.05% trypsin-EDTA (GIBCO), centrifuged at 300 g and suspended in 1 ml HPSS. Cell suspension samples were analyzed using a NucleoCounter system (NucleoCounter NC202; hemoMetec A/S) with Via2-Cassettes (Thermo Fisher Scientific Inc.) which contains immobilized acridine orange (AO) and 4′, 6-diamidino-2-phenylindole (DAPI) to distinguish between the total cell and dead cell populations, respectively. The results of the total cell count, and viability percentage were quantified using a NucleoCounter system and viewer (NucleoCounter NC202; hemoMetec A/S). The cell counting and viability assessment were performed with 12 groups × 8 samples/group × 2 time points, with a total of 192 samples, for each parameter. Each experiment was repeated in triplicate for each condition to ensure reliable results (Figure 1B).

### Lactate dehydrogenase (LDH) cytotoxicity analysis

100 μL of the supernatant were collected from each of the 12 test groups (no medication group, 1st medication and the test medication/conc. with and without LPS) were used for LDH analysis (Cytotoxicity Detection Kit LDH; Cat. No. 11 644 793 001; Roche Diagnostics GmbH). This colorimetric cytotoxicity (cell death and cell lysis) assay was used to assess the level of lactate dehydrogenase (LDH) enzyme released from the cells in the supernatant due to plasma membrane damage of the seeded cells at 1- and 3-days intervals. The analysis is based on measuring the LDH-catalyzed conversion of lactate to pyruvate using spectrophotometry. In brief, 100 µl of the supernatant was transferred to a flat-bottom 96-wellplate. Subsequently, 100 µl of a prepared mixture of the catalyst and dye solution was added to the supernatant and the plate was incubated for 30 min at 25°C. The absorbance of the samples was measured at 490 nm using an ELISA reader (xMark, BIO-RAD, Microplate Spectrophotometer). The relative cytotoxicity was then evaluated based on the OD values comparing the different treatment groups against the control (un-treated cells). The lactate dehydrogenase (LDH) assay was performed with 12 groups × 8 samples/group × 2 time points, with a total of 192 samples. Each experiment was repeated in triplicate for each condition to ensure reliable results (Figure 1B).

### IL-1β, Il-6, Il-18, and IgA levels

For the enzyme-linked immunosorbent assay (ELISA), 100 µl of the centrifuged supernatant from each of the 12 groups was utilized. The analysis was performed using a xMark Microplate Spectrophotometer (xMark, BIO-RAD, Microplate Spectrophotometer) and commercially available human ELISA kits (MOLEQULE-ON) designed to measure interleukin-1βeta (IL-1β), IL-6, IL-18, and IgA. Following the manufacturer's guidelines, the ELISA tests were conducted on 96 well plates. The ELISA assay was performed with 12 groups × 8 samples/group at 1 time point with a total of 96 samples for each cytokine analysis. Each experiment was repeated in duplicate for each condition to ensure reliable results (Figure 1B).

### Statistical analysis

Each experiment was repeated in triplicate for each condition to ensure reliable results. Data were analyzed using the Statistical Package for the Social Sciences version 28.0.1 (SPSS Inc.). A two-way ANOVA was employed to analyze the effects of the drugs and the presence of LPS (the two independent variables), followed by Tukey's HSD *post-hoc* test. Paired *t*-tests were conducted to compare data between day 1 (D1) and day 3 (D3). A *p*-value of <0.05 was considered statistically significant. Data are presented in the graphs as means ± standard of error (SE). The assumptions of multiple linear regression were verified by examining the normality of residuals using Q-Q plots and histograms and assessing homoscedasticity through residual vs. predicted value plots. After confirming these assumptions were met, multiple linear regression was conducted to analyze the independent effects of Treatment, LPS exposure, and Day on various outcomes. For each outcome, we estimated a regression model using Treatment (6 levels, with no medication as reference), LPS exposure (No as reference), and Day (Day 1 as reference) as predictors. The resulting regression coefficients quantify the change in the outcome for each level of the factors compared to their respective references. Treatment coefficients indicated the difference in outcome between each treatment and the no medication group, the LPS coefficient showed the effect of LPS exposure, and the Day 3 coefficient revealed the change from Day 1 to Day 3. We tested all possible two-way interactions, Treatment × LPS, Treatment × Day and LPS × Day for their possible inclusion in the models and present the most remarkable model for each of the 9 outcomes.

## Results

### Total cell count

On day 1 for no LPS groups, the Cyclo group showed significant (*p* < 0.002) decreased cell count (231.3 ± 3.03) than all other groups: Ctrl (269.75 ± 2.4), Azi (260.1 ± 4.9), Cyclo: Azi-50(269.75 ± 8.1), Cyclo:Azi-200(255.73 ± 2.8), and Cyclo:Azi-1000(264.66 ± 5.6).

**Figure 2:**
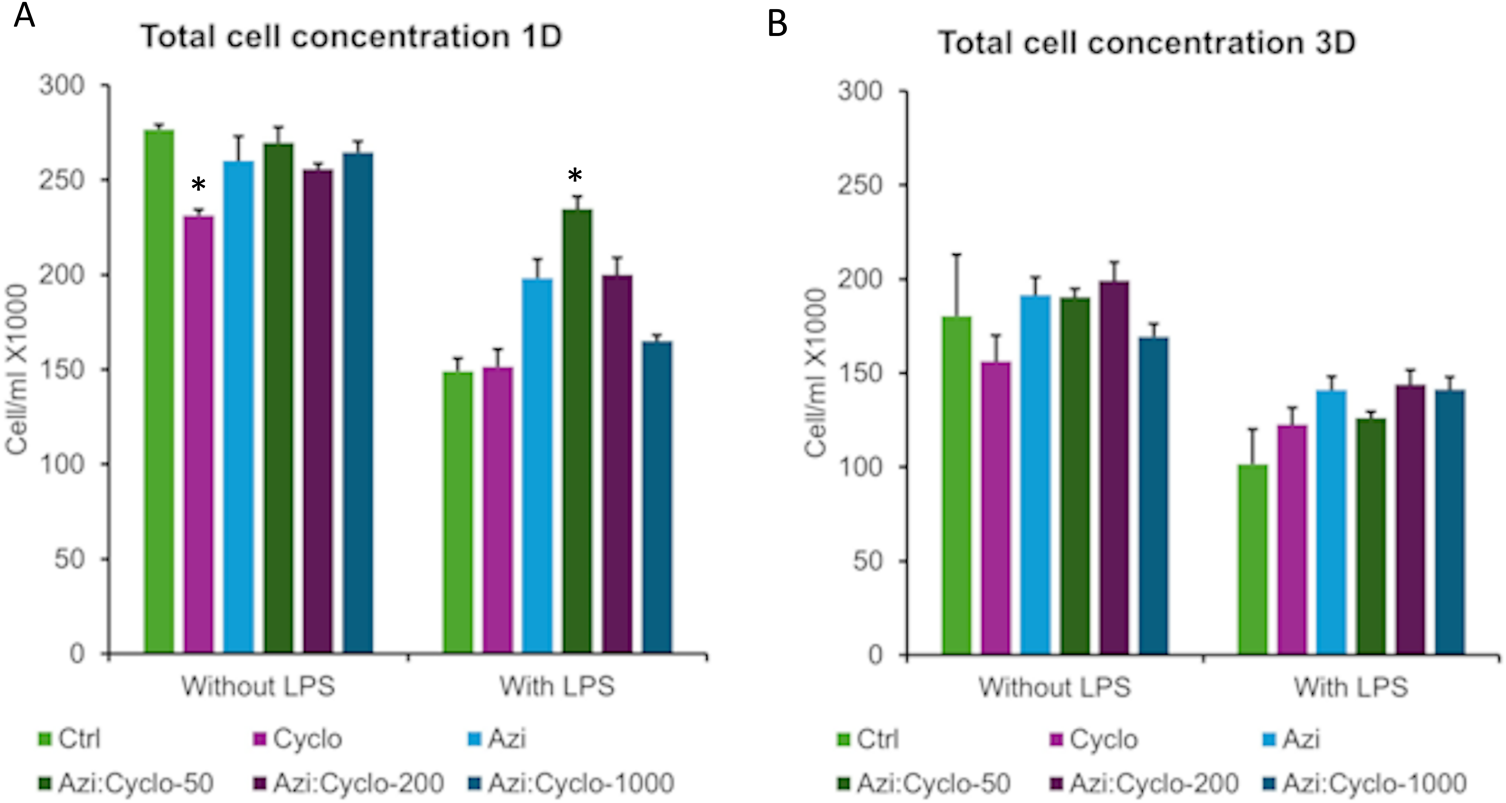
Graphical representation of the total cells counts at day 1 **(A)** and day 3 **(B)** *significant level at *p* < 0.05.

However, with LPS, Cyclo: Azi-50 showed significant more total cells count than all other groups (234.6 ± 6.7, *p* < 0.002). Ctrl (149.1 ± 6.9), Cyclo (151.5 ± 9.1) and Cyclo: Azi-1000(165 ± 3.2) showed significant decrease total cell count than Azi (198.25 ± 9.1), Cyclo: Azi-50 (234.6 ± 6.7), and Cyclo:Azi-200(200 ± 8.9) (*p* = 0.000). Pairwise comparison between no LPS and with LPS showed significant decrease in total cell count for all groups (*p* < 0.042). (Figure 2) The linear regression model showed that none of the treatment groups revealed a significant effect on total count estimate compared to the no-medication reference. However, both LPS exposure and Day 3 assessment significantly reduced cell counts. The model also reveals that the highest reduction in the total cell count estimate is due to the interaction between LPS and day 3 of the assessment (Table 1).

**Table 1:** Multiple linear regression analysis of the effect of treatment, LPS exposure, and day of assessment on total cell count (cell/ml HPSS ×1000).

Variables		B	Sig.	95% CI for B	
				Lower bound	Upper bound
Treatment	No medications (reference)	–	–	–	–
	Cyclosporin 200 ng/ml	−11.56	0.53	−48.62	25.50
	Azithromycin 0.4 μg/ml	20.81	0.26	−16.25	57.87
	Cyclosporin 50 ng/ml + azithromycin 0.4 μg/ml	28.25	0.13	−8.81	65.31
	Cyclosporin 200 ng/ml + azithromycin 0.4 μg/ml	22.69	0.22	−14.37	59.75
	Cyclosporin 1,000 ng/ml + azithromycin 0.4 μg/ml	8.06	0.66	−29.00	45.12
LPS exposure	No (reference)	–	–	–	–
	Yes	−64.13	<0.001*	−81.39	−46.86
Day	Day 1 (reference)	–	–	–	–
	Day 3	−66.17	<0.001*	−83.10	−49.23
Interactions	Cyclosporin 200 ng/ml*LPS	−34.18	0.04*	−66.87	−1.49
	Cyclosporin 50 ng/ml + azithromycin 0.4 μg/ml*day 3	−33.35	0.04*	−66.04	−0.65
	LPS*Day3	−55.88	<0.001*	−78.94	−32.83
Constant	(No medications, No LPS, Day 1)	62.66	0.002*	23.43	101.89

* Significant level at *p* < 0.05.

### Cell viability

The cell viability on day 1 for no LPS groups, ranged between 74% and 88%. Cyclo group showed a significant decrease in cell viability (77.7%) than all other groups (*p* < 0.02). While combining cyclosporin with azithromycin showed comparable values (85%–90%) to the Ctrl (92.2%) group. Under LPS stimulation, the mean viability for all groups dropped to 50%–78% with Cyclo:Azi-50 group showing significantly the highest percentage (78%) (*p* < 0.007). The Cyclo (51%) and the Cyclo: Azi-1000 (55%) showed significantly decreased viability than Azi (66%), Cyclo: Azi1-50 (78%), and Cyclo: Azi-200 (67%) (*p* = 0.000). Pairwise comparison between no LPS and with LPS showed significant decrease in total cell counts as well as cell viability for all groups (*p* < 0.003). The paired *t*-tests comparing the total cell counts for each group from Day 1 to Day 3 showed no significant difference (*p* > 0.05). However, significant decrease in cell viability from Day 1 to Day 3 was observed in Azi: Cyclo-50 (*p* = 0.030), Azi: Cyclo-1000 (*p* = 0.0085), Azi + LPS (*p* = 0.0372), Azi: Cyclo-50+ LPS (*p* = 0.0065), and Azi:Cyclo-200+ LPS (*p* = 0.0436) groups indicating significant changes in these groups over the two days. On day 3, the total cell count was decreased, however no significant difference was observed between the groups with and without LPS stimulation(*p* > 0.05).

**Figure 3:**
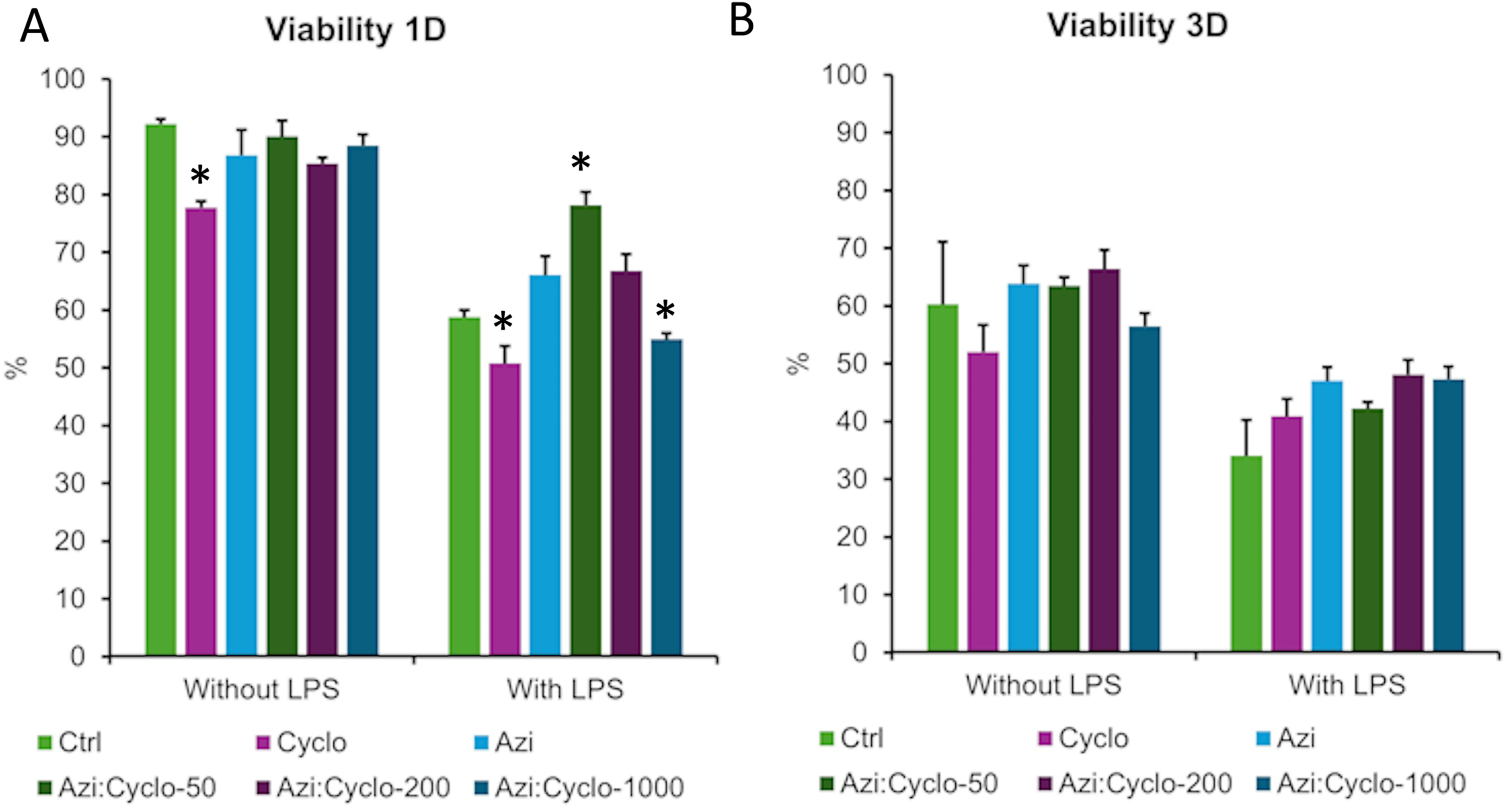
Graphical representation of the viability percentage at day 1 **(A)** and day 3 **(B)** *significant level at *p* < 0.05.

For groups not exposed to LPS the mean viability ranged between 52% and 66%. The lowest viability was observed for the Cyclo group (52%) while the Azi: Cyclo-200 group that showed the highest viability of 66%. For the LPS group the mean viability was decreased ranging from 34% to 48%. Pairwise comparison between no LPS and with LPS showed significant decrease in cell viability for all groups (*P* < 0.033) except Cyclo and Azi: Cyclo-1000 (Figure 3).

The linear regression model showed that both LPS exposure and Day 3 assessment significantly reduced viability. A significant negative interaction was observed between LPS and Day 3 irrespective of treatment group (Table 2).

**Table 2:** Multiple linear regression analysis of the effect of treatment, LPS exposure, and day of assessment on viability percentage (%).

Variables		B	Sig.	95% CI for B	
				Lower bound	Upper bound
Treatment	No-medication (reference)	–	–	–	–
	Cyclosporin 200 ng/ml	−5.96	0.33	−18.23	6.31
	Azithromycin 0.4 μg/ml	4.61	0.45	−7.66	16.87
	Cyclosporin 50 ng/ml + azithromycin 0.4 μg/ml	7.17	0.24	−5.10	19.44
	Cyclosporin 200 ng/ml + azithromycin 0.4 μg/ml	5.34	0.38	−6.92	17.61
	Cyclosporin 1,000 ng/ml + azithromycin 0.4 μg/ml	0.50	0.93	−11.77	12.77
LPS exposure	No (reference)	–	–	–	–
	Yes	−20.68	<0.001*	−26.44	−14.91
Day	Day 1 (reference)	–	–	–	–
	Day 3	−22.85	<0.001*	−28.26	−17.44
Interactions	Cyclosporin 200 ng/ml*LPS	−12.10	0.02*	−22.45	−1.74
	Cyclosporin 200 ng/ml *Day 3	−10.81	0.04*	−21.17	−0.46
	Cyclosporin 50 ng/ml + azithromycin 0.4 μg/ml*Day 3	−11.89	0.02*	−22.25	−1.54
	LPS*Day 3	−18.28	<0.001*	−25.58	−10.98
Constant	(No-medication, No LPS, day 1)	19.79	0.002*	7.36	32.21

* Significant level at *p* < 0.05.

### LDH levels

On day 1 for no LPS groups, the LDH values showed significantly increased values in all groups Cyclo (2.04 ± 0.5), Azi (1.8 ± 0.4), Azi: Cyclo-50 (2.523 ± 0.7), Azi:Cyclo-200 (2.29 ± 0.5), and Azi:Cyclo-1000 (2.430 ± 0.6) compared to the control groups (1.0 ± 0.2) (*p* = 0.042). Similarly, for LPS groups the Ctrl (1.207 ± 0.2) group showed a significant decreased LDH value than Cyclo (2.44 ± 0.2) (*p* < 0.0042), Azi (2.359 ± 1.1), Azi: Cyclo-50 (3.0 ± 0.5), Azi:Cyclo-200 (2.4 ± 1) and Azi:Cyclo-1000 (2.756 ± 0.2) groups.

**Figure 4:**
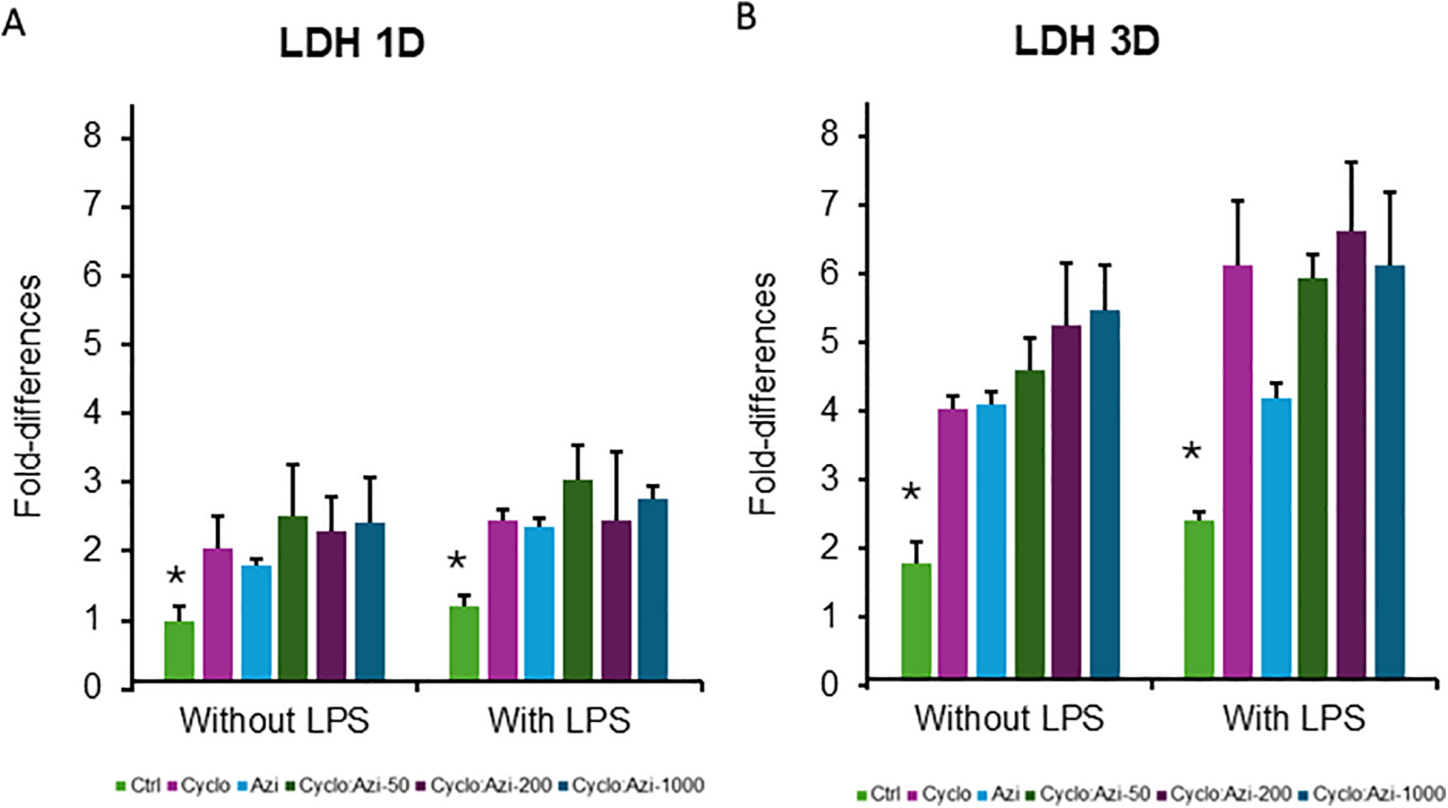
Lactate dehydrogenase (LDH) OD levels for the experimental groups at at day 1 **(A)** and day 3 **(B)** *Significant level at *p* < 0.05.

On day 3 for no LPS groups, the LDH was increased for all groups Cyclo (4.04 ± 0.2), Azi (4.1 ± 0.6), Azi: Cyclo-50 (4.58 ± 0.5), Azi: Cyclo-200 (5.24 ± 0.9), and Azi:Cyclo-1000 (5.47 ± 0.6) showing a significant difference with the Ctrl group (1.79 ± 0.3) (*p* < 0.01). Under LPS stimulation the LDH significantly increased for all groups Cyclo (6.12 ± 0.9), Azi (4.2 ± 0.5), Cyclo-50 (5.95 ± 0.3), Azi: Cyclo-200 (6.6 ± 0.9), and Azi: Cyclo-1000 (6.3 ± 1) compared to the Ctrl (2.42 ± 0.14) (*p* < 0.04), Pairwise comparison between no LPS and with LPS showed no significant difference for LDH both at day-1and day-3 (Figure 4).

The paired *t*-test analysis conducted on the LDH data comparing Day 1 and Day 3 across various test groups. The Cyco group exhibited a highly significant increase in LDH levels from Day 1 to Day 3 (t-statistic = −7.48, *p*-value = 0.005). The linear regression model showed that LPS exposure alone didn't significantly affect LDH levels and Day 3 measurements were significantly higher than Day 1. All treatment groups showed significant positive interactions with Day 3 (Table 3).

**Table 3:** Multiple linear regression analysis of the effect of treatment, LPS exposure, and day of assessment on LDH.

Variables		B	Sig.	95% CI for B	
				Lower bound	Upper bound
Treatment	No-medication (reference)	–	–	–	–
	Cyclosporin 200 ng/ml	0.40	0.002*	0.15	0.66
	Azithromycin 0.4 μg/ml	0.29	0.02*	0.04	0.55
	Cyclosporin 50 ng/ml + azithromycin 0.4 μg/ml	0.47	<0.001*	0.22	0.73
	Cyclosporin 200 ng/ml + azithromycin 0.4 μg/ml	0.52	<0.001*	0.26	0.77
	Cyclosporin 1,000 ng/ml + azithromycin 0.4 μg/ml	0.49	<0.001*	0.24	0.75
LPS exposure	No (reference)	–	–	–	–
	Yes	0.14	0.08	−0.02	0.30
Day	Day 1 (reference)	–	–	–	–
	Day 3	0.50	<0.001*	0.37	0.62
	Cyclosporin 200 ng/ml*day 3	0.48	<0.001*	0.27	0.69
	Azithromycin 0.4 μg/ml*day 3	0.36	0.001*	0.15	0.57
	Cyclosporin 50 ng/ml + Azithromycin 0.4 μg/ml*day 3	0.50	<0.001*	0.29	0.71
	Cyclosporin 200 ng/ml + Azithromycin 0.4 μg/ml*day 3	0.69	<0.001*	0.48	0.90
Interactions	Cyclosporin 1,000 ng/ml + Azithromycin 0.4 μg/ml*day 3	0.62	<0.001*	0.41	0.84
Constant	(No-medication, no LPS, day 1)	−0.19	0.1290	−0.45	0.06

* Significant level at *p* < 0.05.

### IL-6 levels

**Figure 5:**
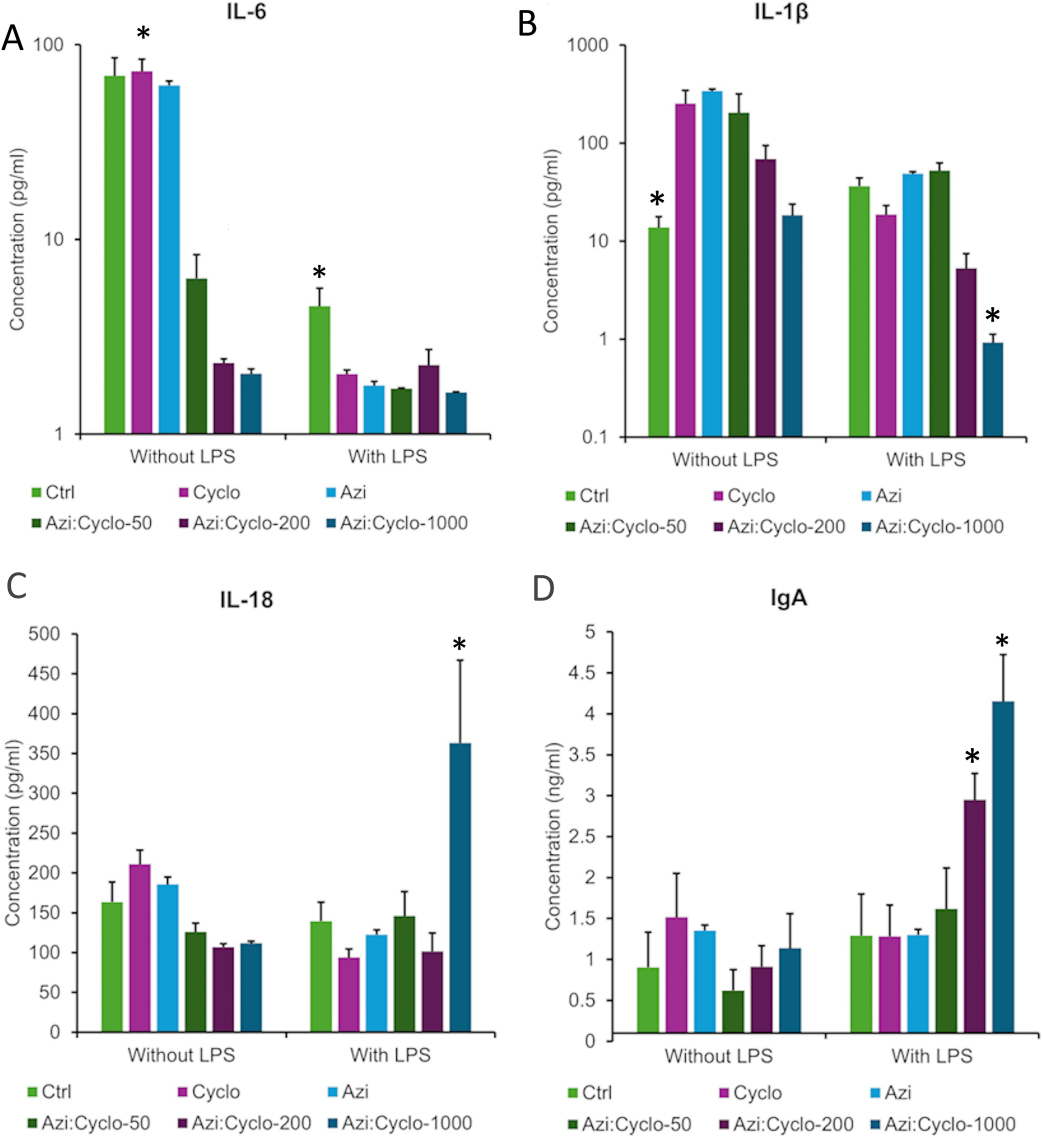
Inflammatory mediators levels assessed by ELISA at day 3 for the experimental groups **(A)** IL-6 pg/ml, **(B)** IL-1β pg/ml, **(C)** IL-18 pg/ml, and **(D)** IgA ng/ml. *Statistically significant difference at *p* value < 0.05.

For no LPS groups, the Ctrl, Cyclo and Azi groups showed comparable levels of IL-6 (69.31 ± 16.4, 73.125 ± 11.3, and 61.95 ± 5.7 pg/ml, respectively). A significant difference was observed between the Azi: Cyclo-50 (6.3 ± 2.0 pg/ml), Azi:Cyclo-200 (2.32 ± 0.12 pg/ml), and Azi:Cyclo-1000 (2.1 ± 0.1 pg/ml) vs. the Ctrl, Cyclo and Azi groups (*p* = 0.000). Under LPS stimulation, all test groups showed decreased IL-6 levels than the Ctrl group (4.54 ± 1.09 pg/ml) suggesting that the addition of LPS significantly reduces IL-6 levels for all treatment groups. The Azi: Cyclo-1000 showed the least IL-6 levels (1.64 ± 0.1 pg/ml) showing significant difference with the Ctrl group (*p* = 0.041). Pairwise comparison between no LPS and with LPS showed significant difference for Ctrl, Cyclo, Azi groups (*p* = 0.000) (Figure 5A).

The linear regression model showed that all combinations of Azi with Cyclo demonstrated significantly lower estimates of IL-6 secretion relative to no-medication. LPS exposure significantly decreased IL-6 secretion, with no significant interactions between treatments and LPS (Table 4).

**Table 4:** Multiple linear regression analysis of the effect of treatment and LPS exposure on IL-6 secretion (pg/ml).

Variables		B	Sig.	95% CI for B	
				Lower bound	Upper bound
Treatment	No-medication (reference)	–	–	–	–
	Cyclosporin 200 ng/ml	0.65	0.96	−26.77	28.08
	azithromycin 0.4 μg/ml	−5.06	0.71	−32.48	22.36
	Cyclosporin 50 ng/ml + azithromycin 0.4 μg/ml	−32.91	0.01*	−60.34	−5.49
	Cyclosporin 200 ng/ml + azithromycin 0.4 μg/ml	−34.63	0.01*	−62.06	−7.21
	Cyclosporin 1,000 ng/ml + azithromycin 0.4 μg/ml	−35.09	0.01*	−62.51	−7.66
LPS exposure	No (reference)	–	–	–	–
	Yes	−33.52	<0.001*	−48.50	−18.54
Interactions	None	–	–	–	–
Constant	(No-medication, No LPS)	44.27	0.002*	16.17	72.36

* Significant level at *p* < 0.05.

### IL-1 β levels

For no LPS groups, the Ctrl group showed the lowest IL-1 beta levels (13.78 ± 4.0 pg/ml) followed by Azi: Cyclo-1000 (18.39 ± 5.5 pg/ml), while the highest levels were observed for Azi (339.89 ± 72.6 pg/ml) followed by the Cyclo (253.49 ± 92.1 pg/ml) groups. A significant difference was observed between Ctrl vs. Cyclo, Azi, and Azi: Cyclo-50 (*p* = 0.003, *p* = 0.002, *p* = 0.007, respectively). A significant difference was observed between Azi: Cyclo-200 (68.79 ± 25.6 pg/ml) and Azi:Cyclo-1000 vs. Cyclo (*p* = 0.012, *p* = 0.003), Azi (*p* = 0.008, *p* = 0.002), and Azi: Cyclo-50 (*p* = 0.029, *p* = 0.008). Under LPS stimulation, the IL-1beta level was decreased for all groups, specially for Azi: Cyclo-1000 (0.92 ± 0.2 pg/ml) followed by Azi: Cyclo-200 (5.29 ± 2.2 pg/ml) then Cyclo (18.7 ± 4.3 pg/ml). The Azi (48.8 ± 35.6 pg/ml) and Azi: Cyclo-50 (52.44 ± 10.3 pg/ml) showed more IL-1beta than the Ctrl (36.44 ± 7.6 pg/ml) group. Azi: Cyclo-1000 showed significant difference with Ctrl (*p* = 0.040), Cyclo (*p* = 0.041), Azi (*p* = 0.040), and Azi: Cyclo-50 (*p* = 0.040) groups. Pairwise comparison between no LPS and with LPS showed significant decrease in IL-1beta for all groups under LPS stimulation (*p* < 0.003) (Figure 5B).

The linear regression model showed that the Azi: Cyclo-200, and Azi: Cyclo-50 treatments without LPS had high IL-1β estimates relative to no-medication. LPS treatment resulted in a significantly lower estimate of IL-1β secretion. Moreover, the LPS interactions with Azi: Cyclo-200 and Azi: Cyclo-1000 were associated with significantly lower estimates of IL-1β secretion (Table 5).

**Table 5:** Multiple linear regression analysis of the effect of treatment LPS exposure on IL-1β secretion (pg/ml).

Variables		B	Sig.	95% CI for B	
				Lower bound	Upper bound
Treatment	No-medication (reference)	–	–	–	–
	Cyclosporin 200 ng/ml	110.99	0.08	−17.72	239.69
	azithromycin 0.4 μg/ml	169.24	0.01*	40.53	297.94
	Cyclosporin 50 ng/ml + azithromycin 0.4 μg/ml	103.50	0.11	−25.20	232.21
	Cyclosporin 200 ng/ml + azithromycin 0.4 μg/ml	11.93	0.85	−116.77	140.64
	Cyclosporin 1,000 ng/ml + Azithromycin 0.4 μg/ml	−15.45	0.80	−144.16	113.25
LPS exposure	No (reference)	–	–	–	–
	Yes	−122.76	0.001*	−195.77	−49.75
Interactions	Cyclosporin 200 ng/ml + azithromycin 0.4 μg/ml*LPS	−148.34	0.04*	−290.19	−6.49
	Cyclosporin 1,000 ng/ml + azithromycin 0.4 μg/ml*LPS	−152.71	0.03*	−294.56	−10.87
Constant	(no-medication, No LPS)	−139.85	0.05	−281.70	2.00

* Significant level at *p* < 0.05.

### IL-18 levels

For no LPS groups, IL-18 levels of the combined cyclosporin and azithromycin groups Azi: Cyclo-50 (126.1 ± 10.7 pg/ml), Azi: Cyclo-200 (106.9 ± 4.2 pg/ml), and Azi: Cyclo-100 (111.6 ± 2.8 pg/ml) showed decrease levels than Ctrl (163.576 ± 25.1 pg/ml), Cyclo (211.023 ± 17.5 pg/ml) and Azi (185.617 ± 19.9 pg/ml) groups.

Under LPS stimulation, IL-18 levels were decreased in Cyclo (94.01 ± 10.5 pg/ml), Azi (122.5 ± 13.2 pg/ml), and Azi: Cyclo-200 (101.6 ± 22.9 pg/ml) groups than the Ctrl (139.489 ± 23.9 pg/ml). The Azi: Cyclo-50 (145.8 ± 30.9 pg/ml) and Azi:Cyclo-1000 (363.313 ± 103.6 pg/ml) showed elevated levels. The elevation in IL-18 levels were significantly in Azi: Cyclo-1000 compared to all other groups (*p* = 0.000). Pairwise comparison between no LPS and with LPS showed that the significant increase under LPS was for Azi: Cyclo-1000 (*p* = 0.000). This suggests that LPS has a markedly different impact on higher concentrations of Cyclosporin compared to the other treatments (Figure 5C).

The linear regression model showed that none of the treatment groups showed significant change in IL-18 secretion relative to the no-medication group. However, considerable positive interaction was demonstrated for LPS with AZI: Cyclo-1000 group revealing the highest IL-18 secretion estimate in the model (Table 6).

**Table 6:** Multiple linear regression analysis of the effect of treatment and LPS exposure on IL-18 secretion (pg/ml).

Variables		B	Sig.	95% CI for B	
				Lower bound	Upper bound
Treatment	No-medication (reference)	–	–	–	–
	Cyclosporin 200 ng/ml	0.98	0.98	−89.63	91.60
	azithromycin 0.4 μg/ml	2.56	0.95	−88.05	93.18
	Cyclosporin 50 ng/ml + azithromycin 0.4 μg/ml	−15.62	0.72	−106.23	75.00
	Cyclosporin 200 ng/ml + azithromycin 0.4 μg/ml	−47.27	0.29	−137.88	43.35
	Cyclosporin 1,000 ng/ml + azithromycin 0.4 μg/ml	85.91	0.06	−4.70	176.53
LPS exposure	No (reference)	–	–	–	–
	Yes	10.34	0.70	−44.88	65.56
Interactions	Cyclosporin 1,000 ng/ml + azithromycin 0.4 μg/ml*LPS	216.54	<0.001*	138.40	294.67
Constant	(No-medication, No LPS)	16.80	0.66	−61.34	94.93

* Significant level at *p* < 0.05.

### IgA levels

For no LPS groups, the IgA level was highest in Cyclo group (1.517 ± 0.5 ng/ml) and lowest for Azi: Cyclo-50 (0.621 ± 0.3 ng/ml). The Azi: Cyclo-200 (0.913 ± 0.3 ng/ml) showed a comparable level to the Ctrl group (0.91 ± 0.4 ng/ml), while the Azi (1.35 ± 0.5 ng/ml) and Azi:Cyclo-1000 (1.14 ± 0.4 ng/ml) levels were more than the Ctrl. No significant difference was observed between the treatment groups without LPS stimulation.

On presence of LPS, IgA levels were increased in all treatment groups however, especially in Azi: Cyclo-200 (2.95 ± 0.3 ng/ml) and Azi: Cyclo-1000 (4.15 ± 0.6 ng/ml) that showed significant difference with Ctrl (1.29 ± 0.5 ng/ml) (*p* < 0.039), Cyclo (1.28 ± 0.4 ng/ml) (*p* < 0.023) and Azi (1.3 ± 0.4 ng/ml) (*p* < 0.027) groups. Azi: Cyclo-1000 was also significantly increased than Azi:Cyclo-50 (*p* = 0.002). Pairwise comparison between no LPS and with LPS showed that the significant increase under LPS was for Azi: Cyclo-200 (*p* = 0.009) and Azi: Cyclo-1000 (*p* = 0.000) (Figure 5D).

The linear regression model showed that Azi: Cyclo-1000 had significantly higher IgA secretion with reference to the no-medication and that LPS exposure increased IgA secretion (Table 7).

**Table 7:** Multiple linear regression analysis of the effect of treatment and LPS exposure on IgA secretion (ng/ml).

Variables		B	Sig.	95% CI for B	
				Lower bound	Upper bound
Treatment	No-medication (reference)	–	–	–	–
	Cyclosporin 200 ng/ml	0.30	0.60	−0.87	1.48
	azithromycin 0.4 μg/ml	0.23	0.69	−0.95	1.40
	Cyclosporin 50 ng/ml + azithromycin 0.4 μg/ml	0.02	0.97	−1.15	1.20
	Cyclosporin 200 ng/ml + azithromycin 0.4 μg/ml	0.83	0.16	−0.34	2.01
	Cyclosporin 1,000 ng/ml + azithromycin 0.4 μg/ml	1.55	0.01*	0.37	2.72
LPS exposure	No (reference)	–	–	–	–
	Yes	1.02	0.002*	0.37	1.68
Interactions	Cyclosporin 200 ng/ml + azithromycin 0.4 μg/ml*LPS	1.81	<0.001*	0.87	2.74
	Cyclosporin 1,000 ng/ml + azithromycin 0.4 μg/ml*LPS	3.01	<0.001*	2.08	3.95
Constant	(No-medication, No LPS)	−0.24	0.61	−1.17	0.70

* Significant level at *p* < 0.05.

## Discussion

This study was conducted to evaluate the effect of combining azithromycin with different concentrations of cyclosporin on peripheral blood mononuclear cells with and without bacterial LPS. None of the treatment groups significantly affected total cell count compared to no-medication group. However, both LPS exposure and Day 3 assessment significantly reduced cell counts and viability. Regarding cell counting and viability percentage, our findings revealed that azithromycin alone or combined with cyclosporin showed better cellular and less cytotoxic effect than cyclosporin alone. However, cyclosporin at higher doses >200 ng/ml even combined with azithromycin exhibited reduction in cell viability, which aligns with its immunosuppressive properties and potential cytotoxicity at higher concentrations. A Previous study by Amsden (22) showed that azithromycin exhibited minimal cytotoxicity, maintains high cell viability and does not induce significant LDH release in human monocytes and macrophages, even at higher doses. Moreover, azithromycin has been shown to have cytoprotective effects, as it reduces cell apoptosis and LDH release in airway epithelial cells exposed to inflammatory stimuli (23).

All treatment groups showed increased LDH levels compared to no-medication and Day 3 measurements were significantly higher than Day 1, with the strongest interaction observed in the cyclosporin 200 ng/ml + azithromycin group. These findings align with Roy et al. (24), who observed time-dependent loss of cell viability and increased toxicity in monocytes. They also observed concentration-dependent effects on cellular viability and higher lactate dehydrogenase (LDH) release with higher doses. These dose-dependent changes were also observed in other studies (25, 26) on PBMCs; however, one study used a different method to measure cell viability, and another used much higher concentrations of cyclosporin.

Cyclosporin has been widely reported to induce dose-dependent cytotoxicity in immune cells. Previous studies (27, 28) have shown that cyclosporin particularly at higher concentrations (>200 ng/ml) increases LDH release and reduces cell viability in PBMCs, due to its effects on mitochondrial function and membrane integrity. Cyclosporin main function is suppression the T cells activation and proliferation, also it disrupts the calcium signal in immune cells particularly in T cells and monocytes, leading to cell death and reduced viability (29).

In the current study, the selection of the cyclosporin concentration was in accordance with the clinical investigation conducted by Daley et al. (30) which demonstrated that patients with mean serum threshold concentrations exceeding 155 ng/ml displayed mild gingival hyperplasia. Furthermore, a broad range of serum concentrations of cyclosporin (50–1,000 ng/ml) are utilized in clinical practice (31). For this investigation, we used the lowest (50 ng/ml) and highest (1,000 ng/ml) of these concentrations, as well as the concentration (200 ng/ml) at which gingival overgrowth begins to be clinically documented.

In this study, azithromycin was administered at a concentration of 0.4 µg/ml, which corresponds to a plasma concentration equivalent to a 500 mg oral dosage (32). This is in accordance with previous clinical trials (21, 32) demonstrating favorable outcomes when targeting this specific plasma concentration. Most studies of combination therapy used a consistent dosage of 500 mg of azithromycin, although the amounts of cyclosporin used have varied significantly. A systematic review concluded that 500 mg of azithromycin administered orally for 5–7 days or for 1-month local application as 85 mg azithromycin per gram of tooth paste significantly reduced cyclosporine-induced gingival enlargement and associated bleeding on probing, although it did not show a significant effect on plaque index and probing depth (3). Argani et al. (33) conducted a randomized controlled trial and revealed that azithromycin-containing toothpaste (85 mg azithromycin per gram of toothpaste) used for 1 month is an effective, simple, and non-invasive treatment for cyclosporine-induced gingival enlargement compared to control group who used placebo toothpaste. Another randomized controlled trial conducted by Abolesaad et al. (34) and Chand et al. (35) demonstrated that 500-mg azithromycin taken orally for 5-days with oral hygiene instructions in renal transplant patients taking cyclosporine is an effective therapeutic tool for managing gingival enlargement and inflammation. Conversely, a study conducted Mesa et al. (36) showed that a 7-day course of 500 mg azithromycin taken orally did not induce remission of cyclosporine-induced gingival enlargement, however, it showed a positive effect on preventing bacterial superimposition and reducing gingival inflammation. Ramalho et al. (21) showed in his RCT that 500 mg azithromycin taken orally for 3 days combined with oral hygiene program induced a significant reduction in cyclosporine-induced gingival overgrowth in renal transplant patients.

In the current study, 100 ng/ml bacterial lipopolysaccharide was used to stimulate inflammation. Bacterial biofilm plays an important role for the pathogenesis of drug-induced gingival enlargement. High-dose LPS administration (100 ng/ml to 1 µg/ml) is commonly used *in vitro* to mechanistically analyze sepsis and chronic inflammatory diseases as well as to promote inflammatory reactions (37). Previous research has reported that hyperplastic and inflamed gingival tissues are associated with specific macrophage phenotypes that express the pro-inflammatory cytokine IL-1β (38).

IL-6 and IL-1β are both proinflammatory cytokines with established involvement in periodontal disease pathogenesis and drug induced gingival enlargements (39). They are both involved in connective tissue turnover but have an opposing role. IL-1β induces breakdown by production of metalloproteinase while Il-6 reduces this destruction through expression of TIMP (39).

All combinations of azithromycin with cyclosporin demonstrated significantly lower IL-6 secretion compared to control. These findings align with previous studies on rheumatoid arthritis patients (40) and liver transplant recipients (41). However, clinical studies on cyclosporin-induced gingival enlargement have shown mixed results (42, 43). According to Atilla et al. (42), there was a reduction in IL-6 levels in the gingival crevicular fluid of kidney transplant patients with cyclosporin induced gingival enlargement compared to sites with gingivitis, although these patients exhibited significantly higher IL-1β levels at sites with gingival enlargement compared to both sites without enlargement and healthy sites in healthy subjects. In contrast, the study by Gürkan et al. (43) on renal transplant patients reported higher IL-6 levels in gingival crevicular fluid of sites both with and without gingival enlargement compared to healthy subjects, and IL-1β was significantly elevated at sites with gingival enlargement compared to those without and to healthy sites.

For IL-1β, azithromycin alone significantly increased levels, while LPS treatment resulted in reduced secretion. The combinations of azithromycin with cyclosporin (200 and 1,000 ng/ml) showed significantly lower IL-1β secretion with LPS.

IL-18 showed no significant changes across treatment groups or with LPS exposure alone. However, a strong positive interaction was observed between LPS and the azithromycin with cyclosporin 1,000 ng/ml combination. Previous studies support varying effects of immunosuppressive therapy on IL-18 regulation (44, 45).

The marked increase in IL-18 at higher concentrations of cyclosporin (1,000 ng/ml) observed in the current study may indicate that these elevated cyclosporin concentrations might have activated cell types beyond monocytes/macrophages, such as T-lymphocytes, to enhance IL-18 production. It is important to note that the mononuclear cell isolation technique (Ficoll-Paque) used in this study typically yields monocytes at a purity of 70%–80%, with lymphocytes also being present. This composition could influence the overall secretion profile of IL-18, suggesting that both monocytes and lymphocytes contribute to the observed increase in IL-18 levels.

The relationship between cyclosporin and immunoglobulin production appears complex. Our findings of cyclosporin-induced decrease in IgA level, particularly in combination with azithromycin, differ from Chang et al. (46), who reported enhanced IgA synthesis with cyclosporin treatment. This discrepancy might be attributed to differences in experimental conditions, drug concentrations, or cellular mechanisms involved in IgA regulation. The enhanced IgA production we observed with LPS stimulation suggests that inflammatory conditions might alter the immunomodulatory effects of these drugs on B cell function and antibody production.

Cytokines play a key role in the pathogenesis of drug-induced gingival enlargement. In patients taking phenytoin, reduced IgA levels can lead to impaired differentiation of IgA-bearing B cells, which may contribute to immune dysregulation and increased gingival mass cells (47). It was reported that drug induced gingival enlargement affects the mechanisms of the host's immune response, resulting in an increase in gingival mass, and that long-term use of these medications could give rise to a decrease in serum and salivary IgA level, inducing periodontal inflammation (48).

During periodontitis, IL-6 and IL-1β have an opposite role in connective tissue turnover. IL-1β promotes tissue breakdown through metalloproteinase production, while IL-6 limits destruction via TIMP expression (39). In periodontitis patients, also elevated levels of IL-18 were observed, that exhibit proinflammatory and chemotactic activities which promote neutrophil activation (44, 45, 49, 50). The observed decrease in these inflammatory mediators levels by azithromycin, particularly without LPS stimulation, suggests its potential therapeutic use in managing cyclosporin-induced gingival enlargement.

The combination of azithromycin with cyclosporin showed complex effects on inflammatory mediators, supporting cyclosporin's broader immunomodulatory effects beyond T lymphocytes (51). Our findings of reduced cytokine production under LPS stimulation further support cyclosporin's broader immunomodulatory effects. Clinically, the use of azithromycin either systemic or topical as an adjunctive therapy in managing gingival enlargement in immunosuppressed patients showed promising results.

Kim et al. (52) investigated the effect of azithromycin on gingival fibroblasts isolated from renal transplant patients. They observed that 50 mg/ml of azithromycin combined with 10 ng/ml cyclosporin A has elevated the reduced activities of metalloproteinase (MMP)-1 and MMP-2, blocked the accumulation of total collagen in culture media, decreased type I collagen mRNA levels, and restored MMP-2 mRNA levels to control values. These findings suggest that azithromycin may improve cyclosporine-induced gingival overgrowth by inhibiting cell proliferation and collagen synthesis, and by activating MMP-2 in gingival fibroblasts. Therefore, combining the two medications may provide a synergistic effect in managing cyclosporine-induced gingival overgrowth by balancing immunosuppression and reducing inflammatory reactions. The British Thoracic Society recommends a long-term, subantimicrobial dose of azithromycin (250–500 mg thrice weekly for 6–12 months) for adult respiratory diseases (53). In periodontal therapy, clinical trials (33–36) have demonstrated the efficacy of 500 mg azithromycin administered orally for 3–7 days, as well as topically (e.g., in toothpaste) for 1 month. However, to minimize the potential risk of antimicrobial resistance and gastrointestinal side effects particularly in patients on immunosuppressants requiring longer-term therapy—locally applied azithromycin with subantimicrobial dose (e.g., in mouthwash or gel or toothpaste) may provide a suitable alternative with fewer systemic side effects (54). Further studies on these topical application methods are recommended.

The clinical relevance of combining azithromycin with cyclosporin lies in its potential to reduce cyclosporin induced gingival overgrowth, that could be attributed to its antibiotic effect, eliminating oral bacteria, reducing local inflammation, and decreasing the extracellular matrix by fibroblasts (21). Our findings demonstrate that lower cyclosporin concentrations (50 and 200 ng/ml) combined with azithromycin preserved cell viability and reduced cytotoxicity compared to higher cyclosporin concentrations (1,000 ng/ml). The 200 ng/ml cyclosporin-azithromycin combination showed optimal results, reducing pro-inflammatory cytokines (IL-6 and IL-1β) and enhancing immune response modulation, as evidenced by elevated IgA levels under LPS stimulation. However, the use of azithromycin, a broad-spectrum antibiotic, raises concerns about its impact on the microbiome and the potential for developing antibacterial resistance. The combination demonstrates better efficacy compared to conventional cyclosporin concentrations, its ability to modulate inflammation and potentially reduce cyclosporin doses necessitate further investigation. The short-term or local drug delivery of azithromycin may be a viable clinical strategy for managing cyclosporin-induced gingival overgrowth, provided that the risks of adverse effects and antibiotic resistance are carefully managed.

To the best of our knowledge, this is the first *in vitro* study examining the effects of azithromycin and cyclosporin on PBMCs. Differences observed between our findings and previously reported ones could be attributed to variations in drug dosages, differences in experimental methodologies, and study design.

The exact explanation for the observed low cytokine levels in the LPS group cannot be identified. Nevertheless, we would like to emphasize that when considering the control groups (without cyclosporin and/or azithromycin), the IL-6 revealed a lower level in the LPS group than non-LPS. In contrast, the IL-1β level was relatively higher in the control-LPS group (36.4 pg/ml) compared to the control-non-LPS group (13.7 pg/ml). When considering the medication groups (treated with cyclosporin and/or azithromycin), a plausible reason for the reduced IL-6 and IL-1β levels under LPS stimulation could be due to interactions of these medications with LPS resulting in an overall immunomodulatory effect on the mononuclear cells, at least at the 3-days evaluation period.

Our findings showed that azithromycin, when combined with cyclosporin at 200 ng/ml, showed beneficial effects by reducing pro-inflammatory cytokines and enhancing immune response while preserving cell viability. However, exposure to LPS significantly impacted cell viability and cytokine production, particularly at higher cyclosporin concentrations. This emphasizes the need for meticulous plaque control and patient education as integral components of periodontal therapy for cyclosporin-induced gingival overgrowth. Among potential treatment options, azithromycin has demonstrated a significant reduction in gingival overgrowth in patients treated with cyclosporine A as well as statistically significant reduction in bleeding on probing (33, 34).

The study limitations include the short follow-up period of only three days. A longer duration is required to explore the long-term effects of these medications. Also, the study focused on undifferentiated monocytes, future studies should incorporate the differentiation of monocytes into macrophages using M-CSF to better understand the effects of azithromycin on cytokine production and anti-inflammatory responses in a more physiologically relevant cell type. Future experiments should include positive controls in LDH assay to establish a baseline for 100% cytotoxicity and validate assay performance. Additionally, investigating the effects of these medications on fibroblast could provide further understandings into their impact on tissue healing and regeneration to enhance their clinical applications.

Given the constraints of systemic azithromycin use that are typically limited to short treatment courses, alternative dosing strategies and minimum inhibitory concentrations should be investigated for local drug delivery approach. This could optimize therapeutic outcomes by reducing gingival inflammation while minimizing the systemic exposure to antibiotics.

In conclusion, the combination of azithromycin with an intermediate cyclosporin dose (200 ng/ml) effectively modulated inflammation while preserving cell viability. This suggests a potential therapeutic approach for managing cyclosporin-induced inflammatory conditions. Further clinical research is needed to establish dosing guidelines and assess long-term efficacy.

## Data Availability

The original contributions presented in the study are included in the article/Supplementary Material, further inquiries can be directed to the corresponding author.
